# Supplementary material for: Invasiveness, Monitoring and Control of Hakea sericea: A Systematic Review
Source: Plants (Basel). 2023 Feb 7;12(4):751. doi: 10.3390/plants12040751 (PMC9963047; doi:10.3390/plants12040751)
Supplement: Supplementary file 1 [file plants-12-00751-s001.zip › plants-2144739-supplementary.pdf]

Supplementary Material (Table S1 and List S1)

Table S1. Number (no.) and percentage (%) of selected publications per country and formulated question (Q).

	AU	SA	PT	GB	NZ	CH	AU SA	PT SP	EU	CH AU US SA	SL UK	SP US	GR	AU SA BR CN PN	AU US	AU SA UK US	AU ZI	AUS BR CH	SA FR NZ	AU NZ	AU SW	US	SP	FR	TOT
Q 1	67	7	3	6	0	3	3	0	0	1	1	1	1	1	1	0	0	1	0	1	1	0	0	0	98
Q 2	16	2	1	0	4	0	0	0	0	0	0	0	0	0	0	0	0	0	1	0	0	0	0	0	24
Q 3	2	39	6	0	1	0	0	1	2	0	0	0	0	0	0	0	0	0	0	0	0	1	1	1	54
Q 1,2	10	0	0	1	0	0	1	0	0	0	0	0	0	0	0	0	0	0	0	0	0	0	0	0	12
Q 1,3	4	4	1	1	1	0	0	0	0	0	0	0	0	0	0	1	1	0	0	0	0	0	0	0	13
Q 1,2,3	2	1	1	1	0	0	0	1	0	0	0	0	0	0	0	0	0	0	0	0	0	0	0	0	6
TOT (no.)	101	53	12	9	6	3	4	2	2	1	1	1	1	1	1	1	1	1	1	1	1	1	1	1	207
%	48,8	25,6	5,8	4,4	2,9	1,5	1,9	1,0	1,0	0,5	0,5	0,5	0,5	0,5	0,5	0,5	0,5	0,5	0,5	0,5	0,5	0,5	0,5	0,5	100,0

AU Australia, SA South Africa, PT Portugal, GB Global, NZ New Zealand, CH Chile, SP Spain, EU Europe, US United States, GR Germany, CN China, ZI Zimbabwe, FR France, SW Swiss.

List S1. Full list of publications selected in the systematic literature search.

Adair, R.J. Biological control of Australian native plants, in Australia, with an emphasis on Acacias. *Muelleria* **2008**, *26*, 67–78. doi: 10.5962/p.292495

Alvarez-Taboada, F.; Paredes, C.; Julián-Pelaz, J. Mapping of the invasive species *Hakea sericea* using Unmanned Aerial Vehicle (UAV) and Worldview-2 Imagery and an object-oriented approach. *Remote Sens.* **2017**, *9, 913.* doi:10.3390/rs9090913

Arnaud, A.; Chapman, D.; Le Roux, J.; Linnamagi, M.; Marchante, E.; Pasiecznik, N.; Pescott, O.; Singh, I.; Starfinger, U.; Vicente, J.; Tanner, R. *Hakea sericea* Schrad. & J.C.Wendl. *EPPO Bull.* **2019**, *49*, 273–279, doi:10.1111/epp.12527

Barker, W. R. Novelties and taxonomic notes relating to *Hakea* Sect. *Hakea* (Proteaceae), mainly of eastern Australia. *J. Adelaide Bot. Gard.* **1996**, *17*, 177-209.

Bell, D. T. Interaction of fire, temperature and light in the germination response of 16 Species from the *Eucalyptus marginata* forest of south-western. *Aust. J. Bot*, **1994**, *42*, 501-509. doi:10.1071/BT9940501

Bell, D.T. Ecological Response Syndromes in the flora of southwestern Western Australia: fire resprouters versus reseeders. *Bot. Rev.* **2001**, *67*, 417–440. doi:10.1007/BF02857891

Bell, D.T.; Van Der Moezel, P.G.; Delfs, J.C.; Loneragan, W.A. Northern sandplain Kwongan: Effect of fire on *Hakea obliqua* and *Beaufortia elegans* population structure. *J. - R. Soc. West. Aust.* **1987**, *69*, 139–143

Bell, D.T.; Vlahos, S.; Watson, L.E. Stimulation of seed-germination of understorey species of the Northern Jarrah Forest of Western-Australia. *Aust. J. Bot.* **1987**, *35*, 593–599. doi:10.1071/BT9870593

Bell, D.T.; Williams, D.S. Tolerance of Thermal Shock in Seeds. *Aust. J. Bot.* **1998**, *46*, 221–233. doi:10.1071/bt97010

Bradstock, R.A.; Gill, A.M.; Hastings, S.M.; Moore, P.H.R. Survival of serotinous seedbanks during bushfires: comparative studies of *Hakea* species from Southeastern Australia. *Aust. J. Ecol.* **1994**, *19*, 276–282. doi:10.1111/j.1442-9993.1994.tb00490.x

Breytenbach, G.J. Alien control: can we afford to slash and burn *Hakea* in Fynbos Ecosystems? *South African For. J.* **1989**, *151*, 6–16. doi:10.1080/00382167.1989.9630499

Brown, C.L.; Whelan, R.J. Seasonal occurrence of fire and availability of germinable seeds in *Hakea sericea* and *Petrophile sessilis*. *J. Ecol.* **1999**, *87*, 932–941. doi:10.1046/j.1365-2745.1999.00401.x

Brunel, S.; Schrader, G.; Brundu, G.; Fried, G. Emerging invasive alien plants for the Mediterranean basin. *EPPO Bull.* **2010**, *40*, 219–238. doi:10.1111/j.1365-2338.2010.02378.x

Canavan, K.; Canavan, S.; Clark, V.R.; Gwate, O.; Richardson, D.M.; Sutton, G.F.; Martin, G.D. The alien plants that threaten South Africa's mountain ecosystems. *Land* **2021**, *10*, 1–19. doi:10.3390/land10121393

Cardillo, M.; Weston, P.H.; Reynolds, Z.K.M.; Olde, P.M.; Mast, A.R.; Lemmon, E.M.; Lemmon, A.R.; Bromham, L. The phylogeny and biogeography of *Hakea* (Proteaceae) reveals the role of biome shifts in a continental plant radiation. *Evolution (N. Y)* **2017**, *71*, 1928–1943. doi:10.1111/evo.13276

Causley, C.L.; Fowler, W.M.; Lamont, B.B.; He, T. Fitness benefits of serotiny in fire- and drought-prone environments. *Plant Ecol.* **2016**, *217*, 773–779. doi:10.1007/s11258-015-0552-y

Cheney, C.; van Wilgen, N.J.; Esler, K.J.; Foxcroft, L.C.; McGeoch, M.A. Quantifying range structure to inform management in invaded landscapes. *J. Appl. Ecol.* **2021**, *58*, 338–349. doi:10.1111/1365-2664.13765

Clarkson, B.R.; Smale, M.C.; Williams, P.A.; Wiser, S.K.; Buxton, R.P. Drainage, soil fertility and fire frequency determine composition and structure of gumland heaths in northern New Zealand. *N. Z. J. Ecol.* **2011**, *35*, 96–113.

Collins, L.; Boer, M.M.; de Dios, V.R.; Power, S.A.; Bendall, E.R.; Hasegawa, S.; Hueso, R.O.; Nevado, J.P.; Bradstock, R.A. Effects of competition and herbivory over woody seedling growth in a temperate woodland trump the effects of elevated CO2. *Oecologia* **2018**, *187*, 811–823. doi:10.1007/s00442-018-4143-1

Cramer, M.D.; Midgley, J.J. Maintenance costs of serotiny do not explain weak serotiny. *Austral Ecol.* **2009**, *34*, 653–662. doi:10.1111/j.1442-9993.2009.01971.x

Daehler, C.C. Performance comparisons of co-occurring native and alien invasive plants: implications for conservation and restoration. *Annu. Rev. Ecol. Evol. Syst.* **2003**, *34*, 183–211. doi:10.1146/annurev.ecolsys.34.011802.132403

Delgado, M.: Zúñiga-Feest, A.; Alvear, M.; Borie, F. The effect of phosphorus on cluster-root formation and functioning of *Embothrium coccineum* (R. et J. Forst.). *Plant and soil* **2013,** *373*, 765-773. doi: 10.1007/s11104-013-1829-3

Delgado, M.; Suriyagoda, L.; Zúñiga-Feest, A.; Borie, F.; Lambers, H. Divergent functioning of Proteaceae species: the South American *Embothrium coccineum* displays a combination of adaptive traits to survive in high-phosphorus soils. *Funct. Ecol.* **2014**, 28, 1356–1366. doi:10.1111/1365-2435.12303

Delgado, M.; Zúñiga-Feest, A.; Almonacid, L.; Lambers, H.; Borie, F. Cluster roots of *Embothrium coccineum* (Proteaceae) affect enzyme activities and phosphorus lability in rhizosphere soil. *Plant and soil*, **2015**, *395*, 189-200. doi:10.1007/s11104-015-2547-9

Dell, B.; Kuo, J.; Thomson, G.J. Development of proteoid roots in *Hakea obliqua* R. Br.(Proteaceae) grown in water culture. *Aust. J. Bot.* **1980**, 28, 27-37. doi:10.1071/BT9800027

Dennill, G.B. The importance of technique in establishing biocontrol agents—the moth *Carposina autologa* on *Hakea sericea*. *Ann. Appl. Biol.* **1987**, *110*, 163-168. doi: 10.1111/j.1744-7348.1987.tb03243.x

Dennill, G.B.; Gordon, A.J.; Neser, S. Difficulties with the release and establishment of *Carposina autologa* Meyrick (Carposinidae) on the weed *H*akea sericea (Proteaceae) in South Africa. *J. Entomol. Soc. South. Afr.* **1987**, *50*, 463–468.

Dettmann, M.E.; Clifford, H.T. Fossil fruit of the *Grevilleeae* (Proteaceae) in the Tertiary of eastern Australia. *Memoirs of the Queensland Museum* **2005**, *51*, 359-374.

Ding, J.; Travers, S.K.; Eldridge, D.J. Occurrence of Australian woody species is driven by soil moisture and available phosphorus across a climatic gradient. *J. Veg. Sci.* **2021**, 36, e13095. doi: 10.1111/jvs.13095

Dinnage, R.; Skeels, A.; Cardillo, M. Spatiophylogenetic modelling of extinction risk reveals evolutionary distinctiveness and brief flowering period as threats in a hotspot plant genus. *Proc. R. Soc. B: Biol.* **2020**, *287*, 20192817. doi:10.1098/rspb.2019.2817

Ducatillion, C.; Badeau, V.; Bellanger, R.; Buchlin, S.; Diadema, K.; Gild, A.; Thevenet, J. Early detection of invasion risk by exotic plant species introduced in forest arboretum in south-eastern France. Emergence of species of the genus *Hakea*. Measures for management. *Revue d'Ecologie* **2015**, 70, 139-150.

Duncan, C.; Schultz, N.L.; Good, M.K.; Lewandrowski, W; Cook, S. The risk-takers and-avoiders: germination sensitivity to water stress in an arid zone with unpredictable rainfall. *AoB Plants*, **2019**, *11*, plz066. doi:10.1093/aobpla/plz066

Dyer, C.; Richardson, D.M. Population genetics of the invasive Australian shrub *Hakea sericea* (Proteaceae) in South Africa. *S. Afr. J. Bot.* **1992**, *58*, 117-124. doi:10.1016/S0254-6299(16)30881-X

El-Amhir, S.H.; Lamont, B.B.; He, T.; Yan, G. Small-seeded *Hakea* Species tolerate cotyledon loss better than large-seeded congeners. *Sci. Rep.* **2017**, *7*, 1–9. doi:10.1038/srep41520

El-Amhir, S.H.M.; Lim, S.L.; Lamont, B.B.; He, T. Seed Size, Fecundity and Postfire Regeneration Strategy Are Interdependent in Hakea. *PLoS One* **2015**, *10*, 1–12. doi:10.1371/journal.pone.0129027

Enright, N.J.; Goldblum, D. Demography of a non-sprouting and resprouting *Hakea* species (Proteaceae) in fire-prone *Eucalyptus* woodlands of southeastern Australia in relation to stand age, drought and disease. *Plant Ecol*. **1999**, *144*, 71-82. doi:10.1023/A:1009839800864

Erckie, L.; Adedoja, O.; Geerts, S.; Van Wyk, E.; Boatwright, J. S. Impacts of an invasive alien Proteaceae on native plant species richness and vegetation structure. *S. Afr. J. Bot.* **2022**, *144*, 332-338. doi:10.1016/j.sajb.2021.09.017

Esler, K.J.; van Wilgen, B.W.; te Roller, K.S.; Wood, A.R.; van der Merwe, J.H. A Landscape-scale assessment of the long-term integrated control of an invasive shrub in South Africa. *Biol. Invasions* **2010**, *12*, 211–218. doi:10.1007/s10530-009-9443-2

Fischer, M.; Beismann, H. 3D Characterization of the complex vascular bundle system of *Hakea* Fruits based on X-Ray Microtomography (MCT) for a better understanding of the opening mechanism. *Flora Morphol. Distrib. Funct. Ecol. Plants* **2022**, *289*, 1–9. doi:10.1016/j.flora.2022.152035

Forsyth, G.G.; Le Maitre, D.C.; O'Farrell, P.J.; van Wilgen, B.W. The prioritisation of invasive alien plant control projects using a multi-criteria decision model informed by stakeholder input and spatial data. *J. Environ. Manage.* **2012**, 103, 51–57. doi:10.1016/j.jenvman.2012.01.034

Fugler, S.R. Infestations of three Australian *Hakea* Species in South Africa and their control. *South African For. J.* **1982**, *120*, 63–68. doi:10.1080/00382167.1982.9630244

Gordon, A. J.; Fourie, A. Biological control of *Hakea sericea* Schrad. & JC Wendl. and *Hakea gibbosa* (Sm.) Cav.(Proteaceae) in South Africa. *African Entomology*, **2011**, *19*, 303-314. doi:10.4001/003.019.0205

Gordon, A.J. A Review of Established and new insect agents for the biological control of *Hakea sericea* Schrader (Proteaceae) in South Africa. *African Entomol. Mem.* **1999**, *1*, 35–43.

Gordon, A.J. Biology and Host Range of the Stem-Boring Beetle *Aphanasium australe*, a promising agent for the biological control of *Hakea sericea* in South Africa. *BioControl* **2003**, *48*, 113–122. doi:10.1023/A:1021245017334

Gordon, A.J. The impact of the *Hakea* seed-moth *Carposina autologa* (Carposinidae) on the canopy-stored seeds of the weed *Hakea sericea* (Proteaceae). *Agric. Ecosyst. Environ.* **1993**, *45*, 105–113. doi:10.1016/0167-8809(93)90062-T

Gordon, A.J.; Lyons, C.L. Current status of *Carposina autologa* (Lepidoptera: Carposinidae), a biological control agent of silky *Hakea, Hakea Sericea* (Proteaceae) and rock *Hakea, Hakea gibbosa* (Proteaceae) in the Western Cape, South Africa. *African Entomol.* **2017**, *25*, 250–253. doi:10.4001/003.025.0250

Groom, P.K. Implications of terminal velocity and wing loading on *Hakea* (Proteaceae) seed dispersal. *J. R. Soc. West. Aust*. **2010**, *93*, 175.

Groom, P.; Lamont, B. Leaf morphology and life form influence water relations of *Hakea* species on different soil substrates within southwestern Australia. *Acta Oecol* **1995**, 16, 609-620

Groom, P.K.; Lamont, B.B. Ecogeographical analysis of *Hakea* (Proteaceae) in South-Western Australia, with special reference to leaf morphology and life form. *Aust. J. Bot.* **1996**, *44*, 527–542. doi:10.1071/BT9960527

Groom, P.K.; Lamont, B.B. Fruit-Seed relations in *Hakea*: serotinous species invest more dry matter in predispersal seed protection. *Austral Ecol.* **1997**, *22*, 352–355. doi:10.1111/j.1442-9993.1997.tb00682.x

Groom, P.K.; Lamont, B.B. Phosphorus accumulation in Proteaceae Seeds: a synthesis. *Plant Soil* **2010**, *334*, 61–72. doi:10.1007/s11104-009-0135-6

Groom, P.K.; Lamont, B.B. Xerophytic implications of increased sclerophylly: interactions with water and light in *Hakea psilorrhyncha* seedlings. *New Phytol.* **1997**, *136*, 231-237. doi:10.1046/j.1469-8137.1997.00732.x

Groom, P.K.; Lamont, B.B.; Markey, A.S. Influence of leaf type and plant age on leaf structure and sclerophylly in *Hakea* (Proteaceae). *Aust. J. Bot.* **1997**, *45*, 827–838. doi:10.1071/BT96115

Guilherme Pereira, C.; Hayes, P.E.; Clode, P.L.; Lambers, H. Phosphorus Toxicity, not deficiency, explains the calcifuge habit of phosphorus-efficient Proteaceae. *Physiol. Plant.* **2021**, *172*, 1724–1738. doi:10.1111/ppl.13384.

Guilherme Pereira, C.; Hayes, P.E.; O'Sullivan, O.S.; Weerasinghe, L.K.; Clode, P.L.; Atkin, O.K.; Lambers, H. Trait convergence in photosynthetic nutrient-use efficiency along a 2-million year dune chronosequence in a global biodiversity hotspot. *J. Ecol.* **2019**, *107*, 2006–2023. doi:10.1111/1365-2745.13158

Hammill, K.A.; Bradstock, R.A.; Allaway, W.G. Post-fire seed dispersal and species re-establishment in proteaceous heath. *Aust. J. Bot.* **1998**, *46*, 407-419

Hayes, P.E.; Clode, P.L.; Oliveira, R.S.; Lambers, H. "Proteaceae from phosphorus-impoverished habitats preferentially allocate phosphorus to photosynthetic cells: An adaptation improving phosphorus-use efficiency." *Plant, Cell & Environ.* **2018**, 41, 605-619. doi: 10.1111/pce.13124

Hayes, P.E.; Nge, F.J.; Cramer, M.D.; Finnegan, P.M.; Fu, P.; Hopper, S.D.; Oliveira, R.S.; Turner, B.L.; Zemunik, G.; Zhong, H.; et al. Traits related to efficient acquisition and use of phosphorus promote diversification in Proteaceae in phosphorus-impoverished landscapes. *Plant Soil* **2021**, *462*, 67–88. doi:10.1007/s11104-021-04886-0

Heelemann, S.; Proches, Ş.; Rebelo, A.G.; Van Wilgen, B.W.; Porembski, S.; Cowling, R.M. Fire season effects on the recruitment of non-sprouting serotinous Proteaceae in the eastern (bimodal rainfall) fynbos biome, South Africa. *Austral Ecol.* **2008**, *33*, 119-127. doi: 10.1111/j.1442-9993.2007.01797.x

Hoang, S.A.; Lamb, D.; Sarkar, B.; Seshadri, B.; Kit Yu, R.M.; Anh Tran, T.K.; O'Connor, J.; Rinklebe, J.; Kirkham, M.B.; Vo, H.T.; Bohan, N.S. Phosphorus application enhances alkane hydroxylase gene abundance in the rhizosphere of wild plants grown in petroleum-hydrocarbon-contaminated Soil. *Environ. Res.* **2022**, *204*, 1–10. https://doi.org/10.1016/j.envres.2021.111924.

Hoang, S.A.; Lamb, D.; Seshadri, B.; Sarkar, B.; Cheng, Y.; Wang, L.; Bolan, N.S. Petroleum hydrocarbon rhizoremediation and soil microbial activity improvement via cluster root formation by wild proteaceae plant species. *Chemosphere* **2021**, *275*,. doi:10.1016/j.chemosphere.2021.130135

Holmes, P.M.; Marais, C. Impacts of alien plant clearance on vegetation in the mountain catchments of the Western Cape. *South. African For. J.* **2000**, *189*, 113–117. doi:10.1080/10295925.2000.9631286

James, K.; Bradshaw, K. Detecting plant species in the field with deep learning and drone technology. *Methods Ecol Evol.* **2020**, *11*, 1509-1519. doi:10.1111/2041-210X.13473

Jeffrey, D.W. "Phosphate nutrition of Australian heath plants. II. The formation of polyphosphate by five heath species." *Aust. J. Bot.* **1968**, 16, 603-613. doi: http://doi.org/10.1071/BT968060

Johnston, P.R. Potential of fungi for the biological control of some New Zealand Weeds. *New Zeal. J. Agric. Res.* **1990**, *33*, 1–14. doi:10.1080/00288233.1990.10430655

Jordan, G.J.; Carpenter, R.J.; Brodribb, T.J. Using fossil leaves as evidence for open vegetation. *Palaeogeogr. Palaeoclimatol. Palaeoecol.* **2014**, *395*, 168–175. doi:10.1016/j.palaeo.2013.12.035

Kirkpatrick, J.; Gilfedder, L.; Duncan, F.; Wapstra, M. Frequent Planned fire can prevent succession to woody plant dominance in montane temperate grasslands. *Austral Ecol.* **2020**, *45*, 872–879. doi:10.1111/aec.12901

Kluge, R.L.; Gordon, A.J. The Fixed plot survey method for determining the host range of the flowerbud-feeding weevil dicomada rufa, a candidate for the biological control of *Hakea sericea* in South Africa. *BioControl* **2004**, *49*, 341–355. doi:10.1023/B:BICO.0000025384.15189.38

Kluge, R.L.; Marshall, C.R.; Siebert, M.W. Tebuthiuron as a selective herbicide for the control of *Hakea gibbosa* (Proteaceae) in Mountain Fynbos vegetation. *South African For. J.* **1987**, *140*, 35–38. doi:10.1080/00382167.1987.9630067

Kluge, R.L.; Neser, S. Biological control of *Hakea sericea* (Proteaceae) in South Africa. *Agric. Ecosyst. Environ.* **1991**, *37*, 91–113. doi:10.1016/0167-8809(91)90141-J

Kluge, R.L.; Siebert, M.W. Erytenna *Consputa pascoe* (Coleoptera: Curculionidae) as the main mortality factor of developing fruits of the weed, *Hakea sericea* Schrader, in South Africa. *J. Entomol. Soc. South. Afr.* **1985**, *48*, 241–245.

Knox, K.J.; Morrison, D.A. Effects of inter-fire intervals on the reproductive output of resprouters and obligate seeders in the Proteaceae. *Austral Ecol.* **2005**, *30*, 407-413. doi:10.1111/j.1442-9993.2005.01482.x

Knox, K.J.E.; Clarke, P.J. Fire season and intensity affect shrub recruitment in temperate sclerophyllous woodlands. *Oecologia* **2006**, *149*, 730–739. doi:10.1007/s00442-006-0480-6

Knox, K.J.E.; Clarke, P.J. Response of resprouting shrubs to repeated fires in the dry sclerophyll forest of Gibraltar Range National Park. *Proc. Linn. Soc. New South Wales* **2006**, *127*, 49–56.

Kotula, L.; Clode, P.L.; Ranathunge, K.; Lambers, H. Role of roots in adaptation of soil-indifferent Proteaceae to calcareous soils in south-western Australia. *J. Exp. Bot.* **2021**, *72*, 1490–1505. doi:10.1093/jxb/eraa515

Kuo, J.; Hocking, P J.; Pate, J.S. Nutrient reserves in seeds of selected proteaceous species from south-western Australia. *Aust. J. Bot.* **1982**, 30, 231-249. doi: 10.1071/BT9820231

Kuppusamy, T.; Giavalisco, P.; Arvidsson, S.; Sulpice, R.; Stitt, M.; Finnegan, P.M.; ... Jost, R. Lipid biosynthesis and protein concentration respond uniquely to phosphate supply during leaf development in highly phosphorus-efficient *Hakea prostrata*. *Plant Physiol.* **2014**, *166*, 1891-1911. doi:10.1104/pp.114.248930

Kuppusamy, T.; Hahne, D.; Ranathunge, K.; Lambers, H.; Finnegan, P.M. Delayed greening in phosphorus-efficient *Hakea prostrata* (Proteaceae) is a photoprotective and nutrient-saving strategy. *Funct. Plant Biol.* **2020**, *48*, 218-230. doi:10.1071/FP19285

Lambers, H.; Cawthray, G.R.; Giavalisco, P.; Kuo, J.; Laliberté, E.; Pearse, S.J.; Scheible, W.R.; Stitt, M.; Teste, F.; Turner, B.L. Proteaceae from severely phosphorus-impoverished soils extensively replace phospholipids with galactolipids and sulfolipids during leaf development to achieve a high photosynthetic phosphorus-use-efficiency. *New Phytol.* **2012**, *196*, 1098–1108. doi:10.1111/j.1469-8137.2012.04285.x

Lambers, H.; Wright, I.J.; Guilherme Pereira, C.; Bellingham, P.J.; Bentley, L.P.; Boonman, A.; Cernusak, L.A.; Foulds, W.; Gleason, S.M.; Gray, E.F.; et al. Leaf manganese concentrations as a tool to assess belowground plant functioning in phosphorus-impoverished environments. *Plant Soil* **2021**, *461*, 43–61. doi:10.1007/s11104-020-04690-2

Lamont, B. Factors affecting the distribution of proteoid roots within the root systems of two *Hakea* species. *Aust. J. Bot.* **1973**, *21*, 165–187. doi:10.1071/BT9730165

Lamont, B. The Effect of soil nutrients on the production of proteoid roots by *Hakea* Species. *Aust. J. Bot.* **1972**, *20*, 27–40, doi:10.1071/BT9720027

Lamont, B. The effects of seasonality and waterlogging on the root systems of a number of *Hakea* Species. *Aust. J. Bot.* **1976**, *24*, 691–702. doi:10.1071/BT9760691

Lamont, B.B. Structure, Ecology and physiology of root clusters - a review. *Plant Soil* **2003**, *248*, 1–19. doi:10.1023/A:1022314613217

Lamont, B.B.; Groom, P.K. Green cotyledons of two *Hakea* Species control seedling mass and morphology by supplying mineral nutrients rather than organic compounds. *New Phytol.* **2002**, *153*, 101–110. doi:10.1046/j.0028-646X.2001.00300.x

Lamont, B.B.; Groom, P.K. Seed and Seedling biology of the woody-fruited Proteaceae. *Aust. J. Bot.* **1998**, *46*, 387–406. doi:10.1071/BT96135

Lamont, B.B.; Groom, P.K.; Cowling, R.M. High leaf mass per area of related species assemblages may reflect low rainfall and carbon isotope discrimination rather than low phosphorus and nitrogen concentrations. *Funct. Ecol*. **2002**,*16*, 403-412. doi:10.1046/j.1365-2435.2002.00631.x

Lamont, B.B.; Groom, P.K.; Richards, M.B.; Witkowski, E.T.F. Recovery of *Banksia* and *Hakea* communities after fire in mediterranean Australia - the role of species identity and functional attributes. *Divers. Distrib*. **1999**, *5*, 15-26. doi:10.1046/j.1472-4642.1999.00032.x

Lamont, B.B.; Groom, P.K.; Williams, M.: He, T. LMA, density and thickness: recognizing different leaf shapes and correcting for their nonlaminarity. *New Phytol*. **2015**, *207*, 942-947. doi:10.1111/nph.13465

Lamont, B.B.; Hanley, M.E.; Groom, P.K.; He, T. Bird pollinators, seed storage and cockatoo granivores explain large woody fruits as best seed defense in *Hakea*. *Perspect. Plant Ecol. Evol. Syst.* **2016**, *21*, 55-77. doi:10.1016/j.ppees.2016.05.002

Lamont, B.B.; He, T.; Lim, S.L. *Hakea*, the world's most *Sclerophyllous* Genus, arose in Southwestern Australian Heathland and diversified throughout Australia over the past 12million years. *Aust. J. Bot.* **2016**, *64*, 77–88. doi:10.1071/BT15134

Lamont, B.B.; He, T.; Yan, Z. Evolutionary history of fire-stimulated resprouting, flowering, seed release and germination. *Biol. Rev.* **2019**, *94*, 903–928. doi:10.1111/brv.12483

Lamont, B.B.; Milberg, P. Removal of the testa during germination or establishment increases germinant mortality, decay and water loss. *Seed Sci. Res.* **1997**, *7*, 245–252. doi:10.1017/s0960258500003597

Lamont, B.B.; Pausas, J.G.; He, T.; Witkowski, E.T.F.; Hanley, M.E. Fire as a selective agent for both serotiny and nonserotiny over space and time. *CRC. Crit. Rev. Plant Sci.* **2020**, *39*, 140–172. doi:10.1080/07352689.2020.1768465

Lamont, B.B.; Pérez-Fernández, M.; Rodríguez-Sánchez, J. Soil bacteria hold the key to root cluster formation. *New Phytol*. 2015, *206*, 1156-1162. doi: 10.1111/nph.13228

Lamont, B.B.; Witkowski, E.T.F.; Enright, N.J. Post-fire litter microsites: safe for seeds, unsafe for seedlings. *Ecology*, **1993**, *74*, 501-512. doi:10.2307/1939311

Le Maitre, D.C.; Krug, R.M.; Hoffmann, J.H.; Gordon, A.J.; Mgidi, T.N. *Hakea sericea*: Development of a model of the impacts of biological control on population dynamics and rates of spread of an invasive species. *Ecol. Modell.* **2008**, *212*, 342–358. doi:10.1016/j.ecolmodel.2007.11.011

Le Maitre, D.C.; Thuiller, W.; Schonegevel, L. Developing an approach to defining the potential distributions of invasive plant species: a case study of *Hakea* Species in South Africa. *Glob. Ecol. Biogeogr.* **2008**, 17, 569–584. doi:10.1111/j.1466-8238.2008.00407.x

Le Maitre, D.C.; Van Wilgen, B.W.; Gelderblom, C.M.; Bailey, C.; Chapman, R.A.; Nel, J.A. Invasive alien trees and water resources in South Africa: case studies of the costs and benefits of management. *For. Ecol. Manage.* **2002**, *160*, 143–159. doi:10.1016/S0378-1127(01)00474-1

López, R.; Cano, F.J.; Martin-StPaul, N.K.; Cochard, H.; Choat, B. Coordination of stem and leaf traits define different strategies to regulate water loss and tolerance ranges to aridity. *New Phytol*. **2021**, *230*, 497-509. doi:10.1111/nph.17185

Lubbe, C.M.; Denman, S.; Cannon, P.F.; Groenewald, J.Z.; Lamprecht, S.C.; Crous, P.W. Characterization of *Colletotrichum* species associated with diseases of Proteaceae. *Mycologia* **2004**, *96*, 1268–1279. doi:10.1080/15572536.2005.11832877

Lubbe, C.M.; Denman, S.; Lamprecht, S.C.; Crous, P.W. Pathogenicity of *Colletotrichum* species to *Protea* cultivars. *Australas. Plant Pathol.* **2006**, *35*, 37–41. doi:10.1071/AP05097

Lux, A.; Kohanová, J.; White, P.J. The secrets of calcicole species revealed. *J. Exp. Bot*. **2021**, *72*, 968-970. doi:10.1093/jxb/eraa555

Lyons, C. L., English, K. F., & Hoffmann, J. H. Research on the biological control of *Hakea sericea* over the past ten years: lessons informing future management of the species in the Western Cape province, South Africa. *Afr. Entomol.* **2021**, 29, 768-774. doi: 10.4001/003.029.0768

Lyons, C.L.; Tshibalanganda, M.; Plessis, A. Du using CT-Scanning technology to quantify damage of the stem-boring beetle, *Aphanasium australe*, a biocontrol agent of *Hakea sericea* in South Africa. *Biocontrol Sci. Technol.* **2020**, *30*, 33–41. doi:10.1080/09583157.2019.1682518

Martins, J.; Richardson, D.M.; Henriques, R.; Marchante, E.; Marchante, H.; Alves, P.; Gaertner, M.; Honrado, J.P.; Vicente, J.R. A multi-scale modelling framework to guide management of plant invasions in a transboundary context. *For. Ecosyst.* **2016**, *3*, 1–14. https://doi.org/10.1186/s40663-016-0073-8.

Mason, R.A.B.; Cooke, J.; Moles, A.T.; Leishman, M.R. Reproductive output of invasive versus native plants. *Glob. Ecol. Biogeogr.* **2008**, *17*, 633–640. doi:10.1111/j.1466-8238.2008.00402.x

Máximo, P.; Ferreira, L.M.; Branco, P.S.; Lourenço, A. Invasive plants: turning enemies into value. *Molecules* **2020**, *25*. doi:10.3390/molecules25153529

McLay, T.G.B.; Bayly, M.J.; Ladiges, P.Y. Is South-Western Western Australia a centre of origin for Eastern Australian taxa or is the Centre an artefact of a method of analysis? A comment on *Hakea* and its supposed divergence over the past 12 million years. *Aust. Syst. Bot.* **2016**, *29*, 87–94. doi: 10.1071/SB16024

Meehan, M.; Keirsten-Wakefield, A.; Cowan, M.; Atkinson, J. Premature opening and dimorphism *in'Hakea decurrens*'(Proteaceae) follicles: A bet-hedging regeneration strategy?. *Vic. Nat*. **2015**, *132*, 139-146.

Meijninger, W.M.L.; Jarmain, C. Satellite-based annual evaporation estimates of invasive alien plant species and native vegetation in South Africa. *Water S.A*. 2014, *40*, 95-108. doi:10.4314/wsa.v40i1.12

Midgley, J. What are the relative costs, limits and correlates of increased degree of serotiny?. *Austral Ecol*. **2000**, *25*, 65-68. doi:10.1046/j.1442-9993.2000.01011.x

Midgley, J.J.; Cowling, R.M.; Lamont, B.B. Relationship of follicle size and seed size in *Hakea* (Proteaceae); isometry, allometry and adaptation. *South African J. Bot.* **1991**, *57*, 107–110. doi:10.1016/s0254-6299(16)30968-1

Milberg, P.; Lamont, B.B. Seed/cotyledon size and nutrient content play a major role in early performance of species on nutrient-poor soils. *New Phytol.* **1997**, *137*, 665–672. doi:10.1046/j.1469-8137.1997.00870.x

Milberg, P.; Pérez-Fernández, M.A.; Lamont, B.B. Seedling growth response to added nutrients depends on seed size in three woody genera. *J. Ecol.* **1998**, *86*, 624-632. doi:10.1046/j.1365-2745.1998.00283.x

Miller, R.G.; Tangney, R.; Enright, N.J.; Fontaine, J.B.; Merritt, D.J.; Ooi, M.K.J.; Ruthrof, K.X.; Miller, B.P. Mechanisms of fire seasonality effects on plant populations. *Trends Ecol. Evol.* **2019**, *34*, 1104–1117. doi:10.1016/j.tree.2019.07.009

Mitchell, D.T.; Allsopp, N. Changes in the phosphorus composition of seeds of *Hakea sericea* (Proteaceae) during germination under low phosphorus conditions. *New Phytologist* **1984**, 96, 239-247. doi: 10.1111/j.1469-8137.1984.tb03560.x

Moll, E.J.; Trinder-Smith, T. Invasion and control of alien woody plants on the Cape peninsula mountains South Africa - 30 Years On. *Biol. Conserv.* **1992**, *60*, 135–143. doi:10.1016/0006-3207(92)91164-N

Moodley, D.; Geerts, S.; Rebelo, T.; Richardson, D.M.; Wilson, J.R.U. Site-specific conditions influence plant naturalization: the case of alien Proteaceae in South Africa. *Acta Oecologica* **2014**, *59*, 62–71. doi:10.1016/j.actao.2014.05.005

Moodley, D.; Geerts, S.; Richardson, D.M.; Wilson, J.R.U. The importance of pollinators and autonomous self-fertilisation in the early stages of plant invasions: *Banksia* and *Hakea* (Proteaceae) as case studies. *Plant Biol.* **2016**, *18*, 124–131. doi:10.1111/plb.12334

Morais, M.C.; Cabral, J.A.; Gonçalves, B. Seasonal variation in the leaf physiology of co-occurring invasive (*Hakea sericea*) and native (*Pinus pinaster*) woody species in a Mediterranean-Type Ecosystem. *For. Ecol. Manage.* **2021**, *480*, 118662. doi:10.1016/j.foreco.2020.118662

Morais, M.C.; Gonçalves, B.; Cabral, J.A. A Dynamic modeling framework to evaluate the efficacy of control actions for a woody invasive plant, *Hakea sericea*. *Front. Ecol. Evol.* **2021**, *9*, 1–9. doi:10.3389/fevo.2021.641686

Moran, V.C.; Hoffmann, J.H. Conservation of the Fynbos Biome in the Cape Floral Region: The role of biological control in the management of invasive alien trees. *BioControl* **2012**, *57*, 139–149. doi:10.1007/s10526-011-9403-5

Morris, M.J. A method for controlling *Hakea sericea* Schrad. seedlings using the fungus *Colletotrichum gloeosporioides* (Penz.) Sacc. *Weed Res.* **1989**, *29*, 449–454. doi:10.1111/j.1365-3180.1989.tb01317.x

Morris, M.J. The use of plant pathogens for biological weed control in South Africa. *Agric. Ecosyst. Environ.* **1991**, *37*, 239–255. doi:10.1016/0167-8809(91)90153-O

Morris, M.J.; Wood, A.R.; Den Breeyen, A. Plant pathogens and biological control of weeds in South Africa: a review of projects and progress during the last decade. *African Entomol.* **1999**, 129–137.

Morrison, D.A.; Renwick, J.A. Effects of variation in fire intensity on regeneration of co-occurring species of small trees in the Sydney Region. *Aust. J. Bot.* **2000**, *48*, 71–79. doi:10.1071/BT98054

Muler, A.L.; Oliveira, R.S.; Lambers, H.; Veneklaas, E.J. Does cluster-root activity benefit nutrient uptake and growth of co-existing species?. *Oecologia*, **2014**, *174*, 23-31. doi:10.1007/s00442-013-2747-z

Nolan, R.H.; Fairweather, K.A.; Tarin, T.; Santini, N.S.; Cleverly, J.; Faux, R.; Eamus, D. Divergence in plant water-use strategies in semiarid woody species. *Funct. Plant Biol*. **2017**, *44*, 1134-1146. doi:10.1071/FP17079

Nunes, L.J.R.; Rodrigues, A.M.; Loureiro, L.M.E.F.; Sá, L.C.R.; Matias, J.C.O. Energy recovery from invasive species: creation of value chains to promote control and eradication. *Recycling* **2021**, *6*, 21. doi:10.3390/recycling6010021

Olde, P. The Hakeinae in horticulture. *Acta Hortic*. **2015**, 1097. doi:10.17660/ActaHortic.2015.1097.18

Oyanoghafo, O.O.; O' Brien, C.; Choat, B.; Tissue, D.; Rymer, P.D. Vulnerability to xylem cavitation of *Hakea* Species (Proteaceae) from a range of biomes and life histories predicted by climatic niche. *Ann. Bot.* **2021**, *127*, 909–918. doi:10.1093/aob/mcab020

Paungfoo-Lonhienne, C.; Lonhienne, T.G.A.; Rentsch, D.; Robinson, N.; Christie, M.; Webb, R.I.; Gamage, H.K.; Carroll, B.J.; Schenk, P.M.; Schmidt, S. Plants can use protein as a nitrogen source without assistance from other organisms. *Proc. Natl. Acad. Sci. U. S. A.* **2008**, *105*, 4524–4529. doi:10.1073/pnas.0712078105

Paungfoo-Lonhienne, C.; Schenk, P.M.; Lonhienne, T.G.; Brackin, R.; Meier, S.; Rentsch, D.; Schmidt, S. Nitrogen affects cluster root formation and expression of putative peptide transporters. *J. Exp. Bot.* **2009**, *60*, 2665-2676.

Pearce, C.A.; Reddell, P.; Hyde, K.D. Revision of the Phyllachoraceae (Ascomycota) on hosts in the angiosperm family, Proteaceae. *Aust. Syst. Bot.* **2001**, *14*, 283–328. doi:10.1071/SB00006

Penman, T.D.; Penman, S.H. Influence of Prescribed burning on fruit production in Proteaceae. *Pacific Conserv. Biol.* **2010**, *16*, 46–53. doi:10.1071/pc100046

Pepo, C.; Forte, P.; Teixeira, G.; Monteiro, A. Gestão das invasoras lenhosas" *Hakea salicifolia*" e "*Hakea sericea*". In Proceedings of the XII Congr. da SEMh/XIX Congr. da ALAM/II Congr. da IBCM, Lisboa (2009).

Pepo, C.; Monteiro, A.; Forte, P.; Teixeira, G. Biologia da germinação das invasoras *Hakea salicifolia* e *H. sericeae*. In Proceedings of the XII Congreso da SEMh/XIX Congreso da ALAM/II Congreso da IBCM, Lisbon, Portugal, 10 November 2009.

Perry, G.L.W.; Wilmshurst, J.M.; McGlone, M.S. Ecology and Long-term history of fire in New Zealand. *N. Z. J. Ecol.* **2014**, *38*, 157–176.

Pickard, J.O.H.N.; Jacobs, S.W.L. *Vegetation Patterns on the Sassafras Plateau*; Australian and New Zealand Geomorphology Group: Wollongong, Australia, 1983; pp. 54–65.

Poot, P.; Lambers, H. Are Trade-Offs in allocation pattern and root morphology related to species abundance? a congeneric comparison between rare and common species in the South-Western Australian Flora. *J. Ecol.* **2003**, *91*, 58–67. doi:10.1046/j.1365-2745.2003.00738.x

Poot, P.; Lambers, H. Growth responses to waterlogging and drainage of woody *Hakea* (Proteaceae) seedlings, originating from contrasting habitats in South-Western Australia. *Plant Soil* **2003**, *253*, 57–70. doi:10.1023/A:1024540621942

Prodhan, M.A.; Jost, R.; Watanabe, M.; Hoefgen, R.; Lambers, H.; Finnegan, P.M. Tight Control of Nitrate Acquisition in a Plant Species That Evolved in an Extremely Phosphorus-Impoverished Environment. *Plant Cell Environ.* **2016**, *39*, 2754–2761,.doi:10.1111/pce.12853

Prodhan, M.A.; Jost, R.; Watanabe, M.; Hoefgen, R.; Lambers, H.; Finnegan, P.M. Tight control of sulfur assimilation: An adaptive mechanism for a plant from a severely phosphorus-impoverished habitat. *New Phytol.* **2017**, *215*, 1068–1079, doi:10.1111/nph.14640

Queirós, C.S.G.P.; Cardoso, S.; Ferreira, J.; Miranda, I.; Lourenço, M.J. V.; Pereira, H. Characterization of Hakea Sericea Fruits Regarding Chemical Composition and Extract Properties. *Waste and Biomass Valorization* **2020**, *11*, 4859–4870. doi:10.1007/s12649-019-00818-3

Rafferty, C.; Lamont, B.B.; Hanley, M.E. Selective feeding by Kangaroos (*Macropus fuliginosus*) on seedlings of Hakea Species: effects of chemical and physical defences. *Plant Ecol.* **2005**, *177*, 201–208. doi:10.1007/s11258-005-2362-0

Rafferty, C.M.; Lamont, B.B.; Hanley, M.E. Herbivore feeding preferences in captive and wild populations. *Austral Ecol.* **2010**, *35*, 257–263. doi:10.1111/j.1442-9993.2009.02031.x

Rathé, A.A.; Pilkington, L.J.; Gurr, G.M.; Daugherty, M.P. Potential for persistence and within-plant movement of *Xylella fastidiosa* in Australian native plants. *Australas. Plant Pathol.* **2012**, *41*, 405–412. doi:10.1007/s13313-011-0116-0

Richards, M.B.; Groom, P.K.; Lamont, B.B. A Trade-off between Fecundity and drought susceptibility in adults and seedlings of *Hakea* Species as influenced by leaf morphology. *Aust. J. Bot.* **1997**, *45*, 301–309. doi:10.1071/BT96012

Richards, M.B.; Lamont, B.B. Post-Fire mortality and water relations of three congeneric shrub species under extreme water stress - a trade-off with fecundity? *Oecologia* **1996**, *107*, 53–60. doi:10.1007/BF00582234

Richardson, D.M. A Cartographic analysis of physiographic factors influencing the distribution of *Hakea* Spp in the South Western Cape. *South African For. J.* **1984**, *128*, 36–40. doi:10.1080/00382167.1984.9628925

Richardson, D.M.; Manders, P.T. Predicting Pathogen-induced Mortality in Hakea Sericea (Proteaceae), an Aggressive Alien Plant Invader in South Africa. *Ann. Appl. Biol.* **1985**, *106*, 243–254. doi:10.1111/j.1744-7348.1985.tb03114.x

Richardson, D.M.; Rejmánek, M. Trees and shrubs as invasive alien species - a global review. *Divers. Distrib.* **2011**, *17*, 788–809. doi:10.1111/j.1472-4642.2011.00782.x

Richardson, D.M.; Van Wilgen, B.W. Factors affecting the regeneration success of *Hakea seri*cea. *South African For. J.* **1984**, *131*, 63–68. doi:10.1080/00382167.1984.9629532

Richardson, D.M.; Van Wilgen, B.W. The Effects of fire in felled *Hakea sericea* and Natural Fynbos implications for weed control in Mountain Catchments. *South African For. J.* **1986**, *139*, 4–14. doi:10.1080/00382167.1986.9630051

Richardson, D.M.; Van Wilgen, B.W.; Mitchell, D.T. Aspects of the reproductive ecology of four australian *Hakea* Species (Proteaceae) in South Africa. *Oecologia* **1987**, *71*, 345–354. doi:10.1007/BF00378706

Roelofs, R.F.R.; Rengel, Z.; Cawthray, G.R.; Dixon, K.W.; Lambers, H. Exudation of carboxylates in Australian Proteaceae: chemical composition. *Plant, Cell & Environ.* **2001**, *24*, 891-904. doi:10.1046/j.1365-3040.2001.00741.x

Sampson, J.F.; Byrne, M.; Gibson, N.; Yates, C. Limiting inbreeding in disjunct and isolated populations of a woody shrub. *Ecol. Evol.* **2016**, *6*, 5867–5880. doi:10.1002/ece3.2322

Santini, N.S.; Cleverly, J.; Faux, R.; Lestrange, C.; Rumman, R.; Eamus, D. Xylem traits and water-use efficiency of woody species co-occurring in the Ti Tree Basin Arid Zone. *Trees - Struct. Funct.* **2016**, *30*, 295–303. doi:10.1007/s00468-015-1301-5

Santini, N.S.; Cleverly, J.; Faux, R.; McBean, K.; Nolan, R.; Eamus, D. Root xylem characteristics and hydraulic strategies of species co-occurring in semi-arid Australia. *IAWA J.* **2018**, *39*, 43-62.

Sañudo, Í.P. Aportaciones a la flora del sur de Galicia (NO España). *Bot. Complut.* **2006**, *30*, 113-116.

Schmidt, S.; Mason, M.; Sangtiean, T.; Stewart, G.R. Do cluster roots of *Hakea* actities (Proteaceae) acquire complex organic nitrogen?. *Plant Soil* **2003**, *248*, 157–165, doi:10.1023/A:1022352415728

Schmidt, S.; Stewart, G.R. Glycine metabolism by plant roots and its occurrence in Australian plant communities. *Funct. Plant Biol.* **1999**, *26*, 253-264. doi:10.1071/PP98116

Schmidt, S.; Stewart, G.R. Waterlogging and Fire Impacts on Nitrogen Availability and utilization in a Subtropical Wet Heathland (Wallum). *Plant, Cell Environ.* **1997**, *20*, 1231–1241. doi:10.1046/j.1365-3040.1997.d01-20.x

Schütte, K.H. *Hakea* Eradication by means of new herbicides. *J. South African For. Assoc.* **1953**, *23*, 30–36. doi:10.1080/03759873.1953.9630722

Shane, M.W.; Cramer, M.D.; Funayama-Noguchi, S.; Cawthray, G.R.; Millar, A.H.; Day, D.A.; Lambers, H. Developmental physiology of cluster-root carboxylate synthesis and exudation in harsh *Hakea*. *Plant Physiol.* **2004**, *135*, 549-560. doi:10.1104/pp.103.035659

Shane, M.W.; De Vos, M.; De Roock, S.; Cawthray, G.R.; Lambers, H. Effects of external phosphorus supply on internal phosphorus concentration and the initiation, growth and exudation of cluster roots in *Hakea prostrata* R.Br. *Plant Soil* **2003**, *248*, 209–219, doi:10.1023/A:1022320416038

Shane, M.W.; Lambers, H. Cluster Roots: A Curiosity in Context. *Plant Soil* **2005**, *274*, 101–125. doi:10.1007/s11104-004-2725-7

Shane, M.W.; Lambers, H. Manganese accumulation in leaves of *Hakea prostrata* (Proteaceae) and the significance of cluster roots for micronutrient uptake as dependent on phosphorus supply. *Physiol. Plant.* **2005**, *124*, 441–450. doi:10.1111/j.1399-3054.2005.00527.x

Shane, M.W.; McCully, M.E.; Lambers, H. Tissue and cellular phosphorus storage during development of phosphorus toxicity in *Hakea prostrata* (Proteaceae). *J. Exp. Bot.* **2004**, *55*, 1033–1044, doi:10.1093/jxb/erh111

Shane, M.W.; Stigter, K.; Fedosejevs, E.T.; Plaxton, W.C. Senescence-inducible cell wall and intracellular purple acid phosphatases: implications for phosphorus remobilization in *Hakea prostrata* (Proteaceae) and *Arabidopsis thaliana* (Brassicaceae). *J. Exp. Bot.* **2014**, *65*, 6097-6106. doi:10.1093/jxb/eru348

Shane, M.W.; Szota, C.; Lambers, H. A root trait accounting for the extreme phosphorus sensitivity of *Hakea prostrata* (Proteaceae). *Plant, Cell & Environ.* **2004**, *27*, 991-1004. doi:10.1111/j.1365-3040.2004.01204.x

Silva, J.S.; Deus, E.; Nereu, M.; Davim, D.; Rossa, C. Aliens & Flames: A new research initiative joining fire behaviour and invasion ecology. In *Advances in Forest Fire Research*, Imprensa da Universidade de Coimbra: Coimbra, Portugal, 2018, Volume 1, pp. 1219–1222. https://doi.org/10.14195/978-989-26-16-506.

Silva, J.S.; Nereu, M.; Queirós, L.; Deus, E.; Fernandes, P. Fire hazard and plant invasions – the cases of *Hakea Sericea* and *Acacia dealbata* in Portugal. In Proceedings of the 15th Conference on Ecology and Management of Alien Plant invasions, Prague, Czech Republic, September of 2019.

Skeels, A.; Cardillo, M. Environmental niche conservatism explains the accumulation of species richness in Mediterranean-hotspot plant genera. *Evolution*, **2017**, *71*, 582-594. doi:10.1111/evo.13179

Skeels, A.; Cardillo, M. Equilibrium and Non-Equilibrium Phases in the Radiation of *Hakea* and the drivers of diversity in Mediterranean-type ecosystems. *Evolution (N. Y)* **2019**, *73*, 1392–1410. doi:10.1111/evo.13769

Skeels, A.; Dinnage, R.; Medina, I.; Cardillo, M. Ecological interactions shape the evolution of flower color in communities across a temperate biodiversity hotspot. *Evol. Lett.* **2021**, *5*, 277–289. doi:10.1002/evl3.225

Smith, L.; Gordon, A.J. A need for an additional biological control agent on *Hakea sericea* Schrad. & J.C. Wendl. (Proteaceae) in South Africa. *Afr. Entomol.* **2009**,*17*, 200–206. doi:10.4001/003.017.0210

Sousa, M.F.; Façanha, A.R.; Tavares, R.M.; Lino-Neto, T.; Gerós, H. Phosphate Transport by Proteoid Roots of *Hakea Sericea*. *Plant Sci.* **2007**, *173*, 550–558. doi:10.1016/j.plantsci.2007.08.006

Sousa, M.F.; Tavares, R.M.; Gerós, H.; Lino-Neto, T. First report of *Hakea sericea* leaf infection caused by *Pestalotiopsis funer*ea in Portugal. *Plant Pathol.* **2004**, *53*, 535. doi:10.1111/j.1365-3059.2004.01042.x

Standish, R.J.; Albornoz, F.E.; Morald, T.K.; Hobbs, R.J.; Tibbett, M. Mycorrhizal symbiosis and phosphorus supply determine interactions among plants with contrasting nutrient-acquisition strategies. *J. Ecol.* **2021**, *109*, 3892–3902. doi:10.1111/1365-2745.13766

Standish, R.J.; Stokes, B.A.; Tibbett, M.; Hobbs, R.J. Seedling response to phosphate addition and inoculation with arbuscular mycorrhizas and the implications for old-field restoration in Western Australia. *Environ. Exp. Bot.* **2007**, *61*, 58–65. doi:10.1016/j.envexpbot.2007.03.004

Stock, W.D.; Pate, J.S.; Delfs, J. Influence of seed size and quality on seedling development under low nutrient conditions in five australian and south african members of the Proteaceae. *J. Ecol.* **1990**, *78*, 1005. doi:10.2307/2260949

Stock, W.D.; Verboom, G.A. Phylogenetic ecology of foliar N and P concentrations and N:P Ratios across Mediterranean-Type ecosystems. *Glob. Ecol. Biogeogr.* **2012**, *21*, 1147–1156. doi:10.1111/j.1466-8238.2011.00752.x

Sulpice, R.; Ishihara, H.; Schlereth, A.; Cawthray, G.R.; Encke, B.; Giavalisco, P.; Ivakov, A.; Arrivault, S.; Jost, R.; Krohn, N.; et al. Low levels of ribosomal RNA partly account for the very high photosynthetic phosphorus-use efficiency of Proteaceae species. *Plant, Cell Environ.* **2014**, *37*, 1276–1298. doi:10.1111/pce.12240

Tanner, R.; Branquart, E.; Brundu, G.; Buholzer, S.; Chapman, D.; Ehret, P.; Fried, G.; Starfinger, U.; van Valkenburg, J. The prioritisation of a short list of alien plants for risk analysis within the framework of the Regulation (EU) No. 1143/2014. *NeoBiota* **2017**, *35*, 87–118. doi:10.3897/neobiota.35.12366

Tasker, E.M.; Denham, A.J.; Taylor, J.E.; Strevens, T.C. Post-fire seed predation: Does distance to unburnt vegetation matter?. *Austral Ecol.* **2011**, *36*, 755-766. doi:10.1111/j.1442-9993.2010.02214.x

Teixeira, G.; Monteiro, A.; Pepo, C. Leaf morphoanatomy in *Hakea sericeae* and *H. salicifolia*. *Microsc. Microanal.* **2008**, *14*, 109–110. doi:10.1017/S1431927608089563

Tonnabel, J.; Van Dooren, T.J.M.; Midgley, J.; Haccou, P.; Mignot, A.; Ronce, O.; Olivieri, I. Optimal resource allocation in a serotinous non-resprouting plant species under different fire regimes. *J. Ecol.* **2012**, *100*, 1464–1474. doi:10.1111/j.1365-2745.2012.02023.x

Van Der Weide, R.Y.; Bleeker, P.O.; Achten, V.T.J.M.; Lotz, L.A.P.; Fogelberg, F.; Melander, B. Innovation in mechanical weed control in crop rows. *Weed Res.* **2008**, *48*, 215–224. doi:10.1111/j.1365-3180.2008.00629.x

Van Rensburg, J.; Van Wilgen, B.W.; Richardson, D.M. Reconstructing the spread of invasive alien plants on privately-owned land in the Cape floristic region: Vergelegen Wine Estate as a Case Study. *South African Geogr. J.* **2018**, *100*, 180–195. doi:10.1080/03736245.2017.1340187

Van Wilgen, B., Richardson, D.M. The effects of alien shrub invasions on vegetation structure and fire behaviour in South African Fynbos Shrublands : a simulation study. J. Appl. Ecol.**1985**, *22*, 955–966.

Van Wilgen, B.W.; Fill, J.M.; Baard, J.; Cheney, C.; Forsyth, A.T.; Kraaij, T. Historical costs and projected future scenarios for the management of invasive alien plants in protected areas in the Cape floristic region. *Biol. Conserv.* **2016**, *200*, 168–177. doi:10.1016/j.biocon.2016.06.008

Van Wilgen, B.W.; Forsyth, G.G.; Le Maitre, D.C.; Wannenburgh, A.; Kotzé, J.D.F.; van den Berg, E.; Henderson, L. An Assessment of the effectiveness of a large, national-scale invasive alien plant control strategy in South Africa. *Biol. Conserv.* **2012**, *148*, 28–38. doi:10.1016/j.biocon.2011.12.035

Van Wilgen, B.W.; Reyers, B.; Le Maitre, D.C.; Richardson, D.M.; Schonegevel, L. A biome-scale assessment of the impact of invasive alien plants on ecosystem services in South Africa. *J. Environ. Manage.* **2008**, 89, 336-349. doi:10.1016/j.jenvman.2007.06.015

Warren, C.R.; Adams, M.A. Capillary electrophoresis for the determination of major amino acids and sugars in foliage: application to the nitrogen nutrition of *Sclerophyllous* species. *J. Exp. Bot.* **2000**, *51*, 1147–1157. doi:10.1093/jexbot/51.347.1147

Wells, M.J. Introduced plants of the fynbos biome. In *Biogeography of Mediterranean Invasions*; Cambridge University Press: Cambridge, United Kingdom, 1991; pp. 115–129.

Whelan, R.J.; York, J. Post-fire germination of *Hakea sericea* and *Petrophile sessili*s after spring burning. *Aust. J. Bot.* **1998**, *46*, 367–376. doi:10.1071/BT97075

Whitworth-Hulse, J.I.; Magliano, P.N.; Zeballos, S.R.; Aguiar, S.; Baldi, G. Global patterns of rainfall partitioning by invasive woody plants. *Glob. Ecol. Biogeogr.* **2021**, *30*, 235–246. doi:10.1111/geb.13218

Williams, P.A. *Hakea Salicifolia*: Biology and role in succession in Abel Tasman National Park, New Zealand. *J. R. Soc. New Zeal.* **1992**, *22*, 1–18. doi:10.1080/03036758.1992.10420814

Williams, P.A. *Hakea sericea*: seed production and role in succession in Golden Bay, Nelson. *J. R. Soc. New Zeal.* **1992**, *22*, 307–320. doi:10.1080/03036758.1992.10420824

Williams, P.R. Clarke, P.J. Habitat segregation by serotinous shrubs in heaths: post-fire emergence and seedling survival. *Aust. J. Bot.* **1997**, *45*, 31-39. doi:10.1071/BT96076

Wood, A.R.; Breeyen, A. Incidence of gummosis disease in silky *Hakea under* natural conditions in South Africa. *South African J. Plant Soil* **2021**, *38*, 126–133. doi:10.1080/02571862.2021.1879286

Wood, A.R.; Den Breeÿen, A. Plant pathogens and biological control of invasive alien plants in South Africa: A Review of Projects and Progress (2011-2020). *African Entomol.* **2021**, *29*, 983–1004. doi:10.4001/003.029.0983

Wyse, S. V.; Perry, G.L.W.; Curran, T.J. Shoot-level flammability of species mixtures is driven by the most flammable species: implications for vegetation-fire feedbacks favouring invasive species. *Ecosystems* **2018**, *21*, 886–900. doi:10.1007/s10021-017-0195-z

Yan, L.; Zhang, X.; Han, Z.; Pang, J.; Lambers, H.; Finnegan, P.M. Responses of foliar phosphorus fractions to soil age are diverse along a 2 Myr dune chronosequence. *New Phytol.* **2019**, *223*, 1621–1633. doi:10.1111/nph.15910

Yusiharni, E.; Gilkes, R. Minerals in the ash of Australian native plants. *Geoderma* **2012**, *189–190*, 369–380. doi:10.1016/j.geoderma.2012.06.035
